# Autoantibodies to Zinc Transporter 8 and *SLC30A8* Genotype in Type 1 Diabetes Childhood: A Pioneering Study in North Africa

**DOI:** 10.1155/2022/2539871

**Published:** 2022-05-23

**Authors:** Raouia Fakhfakh, Sana Kmiha, Safa Tahri, Sawsan Feki, Ferjeni Zouidi, Olfa Abida, Mongia Hachicha, Thouraya Kammoun, Hatem Masmoudi

**Affiliations:** ^1^Autoimmunity, Cancer, And Immunogenetics Research Laboratory, University Hospital Habib Bourguiba of Sfax, Tunisia; ^2^Pediatrics Department, University Hospital Hedi Chaker of Sfax, Tunisia

## Abstract

**Background:**

Type 1 diabetes (T1D) occurs as a result of insulin deficiency due to destructive lesions of pancreatic *β* cells. In addition to classical autoantibodies (Abs) to islet cell antigens, antizinc transporter 8 Abs (ZnT8-Ab) have been recently described in T1D.

**Objective:**

As no data on ZnT8-Ab in Tunisian patients has been reported, we aim to evaluate the relationships between ZnT8-Ab, ZnT8 coding gene (*SLC30A8*) promoter polymorphism, and T1D risk in newly diagnosed children.

**Methods:**

ZnT8-Ab were measured in the serum of T1D newly affected children (*n* = 156) who were admitted to the pediatric department of the Hedi Chaker University Hospital of Sfax. Rs13266634 was genotyped in T1D children and 79 of their first-degree parents. The SPSS software was used to analyze the serological data. Allelic association analysis was conducted with family-based association tests implemented in the FBAT program v1.5.1.

**Results:**

ZnT8-Ab was detected in 66/156 (42.3%) of T1D newly diagnosed children. Among them, 6 (9%) presented ZnT8-Ab as the only humoral marker. The inclusion of ZnT8-Ab increased the number of Ab-positive patients to 90% and reduced the negative ones by 27%. There was no evidence of any overtransmission of any allele of the rs13266634 C/T polymorphism from parents to affected T1D children, nor of any correlation with any clinical or serological parameter. After the T1D disease onset age adjustment, a significant association was observed with the C allele suggesting that it could have a susceptibility role.

**Conclusion:**

ZnT8-Ab appears as a relevant diagnostic marker for T1D in Tunisian children, especially at the onset of the disease as teenagers.

## 1. Introduction

Type 1 diabetes (T1D) is an autoimmune disease resulting from the destruction of *β* islet cells, leading to a lack of insulin production. T1D is characterized by the involvement of cellular immunity, genetic susceptibility, and the presence of specific circulating autoantibodies (-Abs) such as islet cells Abs (ICA), insulin auto-Abs (IAA), glutamic acid decarboxylase Abs (GADA), and antiprotein tyrosine phosphatase- (PTP-) like antigen IA-2-Abs (IA-2A). These auto-Abs to islet cell antigens can be detected by several methods. Albeit not always sufficient to fully predict the disease outcome, they have been demonstrated to be serological markers for the immunological process that precedes the clinical onset of the disease [1–3]. Currently, the zinc transporter 8 protein (ZnT8) has been identified as one of the major autoantigens in T1D humans and was reported to be a predictive marker in risk groups before the onset of the disease [4]. Furthermore, both IA-2A and ZnT8-Ab tend to appear as secondary autoantibodies and seem to be associated with the progression of diabetes [4]. Indeed, Wenzlau et al. followed a group of children from birth to T1D onset. They found that ZnT8-Ab are detected as early as 2 years old and observed that these Abs persist, and their levels increase throughout the disease [5]. The ZnT8-Ab prevalence varies in different populations, being only in 28% of the Japanese patients and much higher in the Caucasian populations (up to 60–80%) [6]. Kawasaki suggested a strong inverse correlation between the age of T1D onset and the prevalence of ZnT8-Ab [7]. A recent study on T1D patients from sub-Saharan Africa (Amhara, the second-largest ethnic group in Ethiopia) showed that the prevalence of ZnT8-Ab is low in all age groups, including those with childhood-onset disease [8].

Unfortunately, the chronological order in the appearance of these four auto-Abs in T1D patients remains unknown, implying that either the initiating antigen is not determined or that there are multiple pathophysiological pathways and diverse forms of human T1D from an immunological standpoint.

Knowing that genetic factors contribute to the incidence rates and clinical characteristics of T1D variation among populations, polymorphisms in *SLC30A8* may affect zinc accumulation in insulin granules and the stability, storage, and secretion of insulin [9].

The *SLC30A8* gene (NC_000008.11), located on chromosome 8q24.11, encodes a ZnT8 protein containing 369 amino acids (aa). Several studies have identified that the major epitopes for ZnT8-Ab are localized in the carboxy-terminal 102 aa (268-369 aa) that lie within the cytoplasmic domain [4, 6]. Furthermore, the common nonsynonymous single nucleotide polymorphism (SNP) rs13266634 causes an amino acid substitution on residue 325 from an arginine (Arg/R), encoded by the C allele, to a tryptophan (Trp/W), encoded by the minor T allele, which is a major determinant [6] and might be critical for humoral autoimmunity in T1D.

To date, the study of ZnT8-Ab as a diagnostic parameter of T1D is still limited in the North Africa region, especially in Tunisia, situated on the Mediterranean coast of North Africa. Therefore, we aimed, first, to evaluate the use of ZnT8-Ab for diagnosing T1D by assessing its prevalence in newly diagnosed Tunisian children with T1D. Second, we intended to investigate the association between the *SLC30A8* rs13266634 polymorphism and T1D in a family-based study and to evaluate several factors in the diagnosis of diabetes aspects by principal component analysis (PCA).

## 2. Methods

### 2.1. Study Populations

The study population enrolled 156 newly diagnosed T1D children admitted to the pediatric department of the Hedi Chaker University Hospital (Sfax, Tunisia), between 2016 and 2020. Eligibility requirements were the age ≤ 16 years at the time of diagnosis of T1D according to the American Diabetes Association (ADA) criteria for diabetes at onset [10], including fasting plasma glucose ≥ 7.0 mmol/l, 2 h postprandial plasma glucose ≥ 11.1 mmol/l, during an oral glucose tolerance test, and classic symptoms of hyperglycemia or hyperglycemic crisis, as well as random plasma glucose concentration of ≥11.1 mmol/l. The diagnosis of T1D was retained on the presence of diabetic ketosis on diagnosis, dependence on insulin therapy for the control of hyperglycemia, and a positive test for at least one of T1D autoantibodies (ICA, GAD, IA2, IAA, and/or ZnT8), since one and usually more of these autoantibodies are present in >90% of individuals when fasting hyperglycemia is initially detected [11, 12]. Children with other types of diabetes were excluded. Thyroid-stimulating hormone (TSH) was used as a screening test to assess thyroid dysfunction in subjects with T1D. The first day of insulin administration was at T1D diagnosis. This cross-sectional 5-year study included all newly diagnosed T1D patients who had blood tests for routine anti-islet cell auto-Abs. Patients with other forms of diabetes were excluded. For the genetic study, the T1D patients along with their 79 first-degree parents not affected by T1D were included. All participants, originating from the south of Tunisia, were asked to sign a consent form according to the study protocol, and all the institutional ethics requirements were met.

### 2.2. Methods

#### 2.2.1. Laboratory Data

Routine anti-islet cell auto-Abs (ICA, GADA, and IA-2A) were performed for every patient on the day of diagnosis, to confirm autoimmune diabetes origin, in the immunology department of the Habib Bourguiba University Hospital (Sfax, Tunisia). GADA and IA-2A were analyzed by enzyme-linked immunosorbent assay (ELISA) commercial kits (EUROIMMUN®, Germany). The upper limit of the normal range was 10 UI/ml for both tests according to the manufacturer's recommendations. The ICA was analyzed using an indirect immunofluorescence test in primate pancreas biochips (EUROIMMUN®, Germany). The pattern of positive control was used as a reference.

#### 2.2.2. ZnT8-Ab Dosage

Serum aliquots were collected from blood samples on the day of diagnosis and stored at -80°C until analysis. The level of ZnT8-Ab was measured in 156 children with newly diagnosed T1D using 2 ELISA commercial kits (Anti-Zinc Transporter 8 ELISA (Medizym®, Germany) and ZnT8 (RSR 97 Ltd., UK)) [3, 13]. These ELISA kits provide *in vitro* quantitative determination of human ZnT8-Ab directed against all three protein isoforms: Arg/R 325, Trp/W 325, and the very rare glutamine 325. They are suitable for more widespread clinical applications [14]. They also have been shown to give a comparable high degree of sensitivity (72% and 76%, respectively), specificity (99% and 97%, respectively), and a low cross-reactivity (0%–3%), according to the proficiency evaluation of assays for auto-Abs to ZnT8 of the Diabetes Antibody Standardization Program (DASP) [15, 16]. The cut-off for positivity was chosen according to the manufacturer's recommendations at ≥15 RU/ml for both kits. The interassay precision was determined by testing fifteen samples, and the %CV reproducibility was 8.7%. The level of ZnT8-Ab was evaluated in sera of 8 unaffected sex- and age-matched children, obtained from the immunology department of the Habib Bourguiba University Hospital (Sfax, Tunisia) and with no family history of T1D, and it was less than the cut-off value of 15 RU/ml for ZnT8-Ab (data not shown). The result of the Abs presence was expressed in ranges and percentages.

#### 2.2.3. Genetic Study

The genetic study was a family study performed on 156 T1D children and their 79 biological first-degree parents. The rs13266634 SNP is the most representative genetic variation located in the promoter region of the *SLC30A8* gene (NG_016991.1) [17]. It was selected using the genotyping data from dbSNP, according to its association with the susceptibility to other autoimmune diseases and for its potential functional relevance. Indeed, this SNP could influence gene expression through changes in the promoter activity. The variant is represented by a C/T variation corresponding to an Arg/Trp aa change in the coding region of the *SLC30A8* gene (aa position 325). Genomic DNA was extracted from whole blood samples using standard proteinase K digestion and a phenol/chloroform extraction procedure. Genotyping was performed using the PCR–RFLP method. Primers were designed using the Primer3 software (http://primer3.ut.ee/). The restriction enzyme was selected using the NEBcutter software (http://nc2.neb.com/NEBcutter2/). The region surrounding the polymorphism was amplified with the following primers: forward, 5′-GGACAGAAAGAGTTCCCATAGCG-3′ and reverse 5′-ATAGCAGCATGTTTGAAGGTGGC-3′. The PCR amplification was carried out in a volume of 25 *μ*l including 1x buffer, 2 mM MgCl_2_, 0.2–0.4 *μ*mol of each primer (Bio Basic®, Canada), 0.12 mM dNTP (Invitrogen®, CA, USA), 1 U Taq polymerase (Invitrogen®, CA, USA), and 50 *η*g of DNA template. Enzymatic digestion was carried out in a total of 10 *μ*l mixture reactions containing 1x buffer, 0.1x BSA, and 2 units of PvuII restriction enzyme (Roche Diagnostics®, Germany). The products were migrated onto a gel composed of 2% agarose and 1% low melting point agarose (UltraPure Low Melting Point Agarose, Invitrogen®, CA, USA) and stained with a GelDoc (Bio-Rad®, USA). The output files were analyzed using the Quantity One software.

### 2.3. Statistical Analysis

The majority of statistical tests were performed using the SPSS software ver.20.0 (SPSS Inc., IL, USA). All data were tested for normality with the Kolmogorov-Smirnov test. Comparisons between the auto-Ab levels were performed using the Chi2 (*χ*2) test or two-sided Fisher's exact test when appropriate, and the correlation with the clinical manifestation and dominant model genotype groups [18] was made by the Pearson test. The differences were considered to be statistically significant if the *p* value was ≤ 0.05. A principal component (PC) analysis was performed with the aim of grouping variables from 156 T1D newly diagnosed children into clusters based on their similarities [19]. Six variables were collected from patients' charts; integrated demographic (gender and age at diagnosis (AAD)), immunological (GADA, IA-2A, and ZnT8-Ab titers), and rs13266634 (predisposing gene variant previously identified as risk factors for autoimmune diabetes) were included in this analysis. For the genetic study, the family-based association test (FBAT) was performed with the FBAT program v1.5.1. This framework uses generalized score statistics to perform a transmission disequilibrium test (TDT) if the dataset consists of parent-affected child trios [20]. The additive and dominant genetic models were applied to each allelic association test, which examines the transmission of the SNP alleles from parents to the affected offspring. The Hardy–Weinberg equilibrium (HWE) was checked in the healthy parents before analysis. AAD adjustments were made using binary logistic regression.

## 3. Results

### 3.1. Patients' Demographic Features

The general characteristics of the 156 patients are presented in Table 1. The sex ratio was 1.1. The average age of AAD was 7.32 ± 4.05 years. Thirty-four patients (21.79%) had a family history of T1D (Table 1). No patient was born from a consanguineous marriage.

### 3.2. Prevalence of ICA, GADA, IA-2A, and ZnT8-Ab

**Figure 1:**
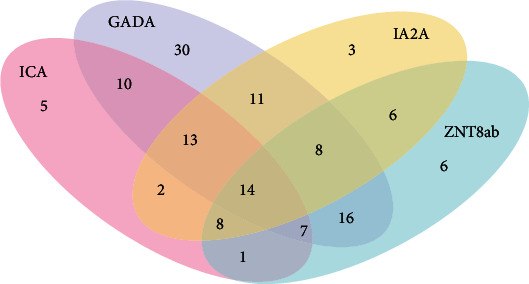
Venn diagrams showing the prevalence and overlap between the prevalence of ICA, GADA, IA-2A, and ZnT8-Ab in type 1 diabetic children. Data showed the number of positive T1D patients. ZnT8-Ab: zinc transporter 8 antibodies; GADA: glutamic acid decarboxylase antibodies; IA-2A: antiprotein tyrosine phosphatase- (PTP-) like antigen IA-2 antibodies.

Substantial overlap was found between ICA, GADA, IA-2A, and ZnT8-Ab and showed that ICA in 35.46%, GADA in 69.87%, ZnT8-Ab in 42.30%, and IA-2A in 41.66% were positive in newly diagnosed T1D children (Figure 1). In this cohort, a positive test for at least one of the three routinely pancreatic Abs (ICA, GADA, and IA-2A) was found in 134 of the 156 children (85.90%). The inclusion of ZnT8-Ab allowed the identification of an additional six patients and increased the prevalence of T1D children presenting at least one Abs to 90%, with a reduction of the number of Abs negative individuals by 27% (Table 2). Regarding single Ab testing, GADA was the most frequently found Ab with 109 T1D children (70%), followed successively by ZnT8-Ab and IA-2A which were positive in 66 (42.30%) and 65 (41.70%) T1D children, respectively, while ICA was detected in 60 of the 156 T1D children (38.5%) (Figure 1).

### 3.3. Correlation between the Presence of ZnT8-Ab and IA-2A

In the 156 T1D newly diagnosed children, only 8.97% were tested positive for all 4 Abs, 23.08% for 3 Abs, 29.49% for 2 Abs specificities, and 28.21% for one Ab, whereas 10.26% of patients were negative for all Abs analyzed. Combining two defined Abs, there was a significantly positive association between IA-2A and ICA (*p* = 0.00001, OR = 3.9, 95% CI (1.97-7.72)). Interestingly, IA-2A was also associated to ZnT8-Ab (*p* = 0.008, OR = 2.52, 95% CI (1.31-4.86)), in addition to the significantly positive correlation between their levels (*p* = 0.004, *r* = 0.234) at T1D onset. Indeed, 56% of the diabetic individuals were positive for ZnT8-Ab compared to 44% of the ZnT8-Ab negative in the positive IA-2A group. However, an inverse trend was found between ZnT8-Ab and GADA (58.71% ZnT8-Ab- compared to 41.28% ZnT8-Ab+ in the GADA^+^ group), with no statistical significance.

### 3.4. The Presence of ZnT8-Ab Is Correlated with AAD but Not with Gender

Globally, there is a significant positive correlation between ZnT8-Ab levels and AAD at T1D onset (*p* = 0.003,*r* = 0.287). Moreover, our study showed that the ZnT8-Ab+ among the T1D patients had a younger AAD than those with ZnT8-Ab- test but without a statistically significant difference (6.26 ± 4.4 vs. 8.20 ± 3.72). This ZnT8-Ab+ group had a significantly higher titer of IA-2A compared to the ZnT8-Ab- group (407.74 ± 86.11 vs. 183.03 ± 63.07, respectively, *p* = 0.037), as well as the same GADA titers (398.66 ± 46.18 vs. 264.89 ± 48.03, respectively). According to the AAD, patients have been divided into two groups: the first group was under 10 years old (children), and the second group was over 10 years old (teenagers). The ICA, GADA, and IA-2A prevalences were similar in the two groups. The ZnT8-Ab, on the other hand, was significantly higher in teenagers than in children (64% vs. 51.88%, *p* = 0.035). This significant association was conserved after AAD adjustment using the binary logistic regression (*p* = 0.004). Moreover, the level of ZnT8-Ab was significantly higher in the teenagers' group, with a mean titer of 225.20 UI/ml, compared to the children's group, with a mean titer of 174.28 UI/ml (*p* = 0.011).

When T1D children were divided into 0-5, 6-10, and 11-14-year-old groups, the significantly higher ZnT8-Ab prevalence in the older T1D patients was maintained (44.64%, 60%, and 64%, respectively, *p* = 0.037). The level of ZnT8-Ab was significantly higher in the 11-14-year-old group, with a mean titer of 362.66 UI/ml, compared to the 0-5-year-old groups, with a mean titer of 129.02 UI/ml (*p* = 0.013) (Table 3). Furthermore, our results showed a nonsignificant difference in the presence and the titers of different Abs according to gender, except for the presence of ICA. Indeed, it was significantly more common in males than in females (62.5% vs. 35.5%, respectively, *p* = 0.045).

### 3.5. Allelic and Genotypic Frequency of the *SLC30A8* Arg325Trp Polymorphism and Its Correlation with Clinical and Biochemical Parameters

The genotype distribution of the *SLC30A8* Arg325Trp polymorphism did not deviate from HWE in the first-degree healthy parents, and the variant allele frequency was 20%. There were no significant differences observed in allele (C: 76% vs. 79% and T: 23% vs. 20%) and genotype (CC: 62% vs. 67%, CT: 27% vs. 24%, and TT: 10% vs. 8%) frequencies of rs13266634 C/T between T1D children and controls, respectively. Upon analysis of 79 parent-offspring trios (one affected child and his two parents) of the study cohort, FBAT analysis identified no over transmission of any allele of the rs13266634 C/T polymorphism from parents to affected T1D children under the additive as well as the dominant models.

To analyze the influence of this common nonsynonymous SNP of the *SLC30A8* gene on clinical and serological parameters, we applied a dominant model comparing the Arg/Arg genotype versus Arg/Trp and Trp/Trp genotypes (named Trp^+^). The results showed that this polymorphism did not affect any parameters. However, Trp^+^ genotype children presented higher ZnT8-Ab titers compared to Arg/Arg genotype children, without statistical significance (178.14 vs. 118.27 U/ml, *p* = 0.6). Moreover, using stepwise logistic regression and after AAD adjustment, a significant association was observed with the C allele (*p* = 0.014, OR = 1.289, 95% CI (1.107-3.780)) which could have a susceptibility role.

**Figure 2:**
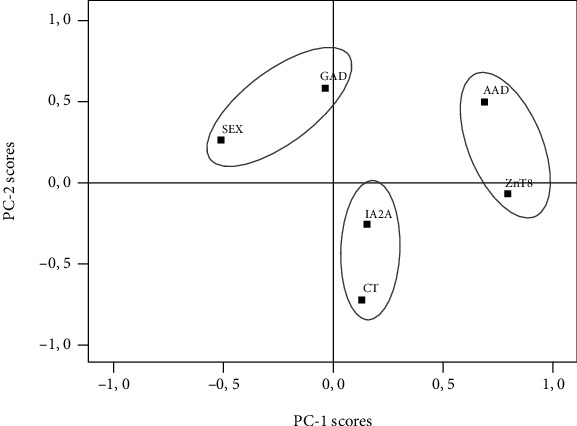
Score plot showing the relationship between the six variables and the two first principal components (PC) of the T1D data set. The 3 circles represent a coherent group, suggesting distinct pathogenic pathways. SEX: gender; GAD: GADA titers; IA2A: IA-2A titers; C/T: rs13266634 genotype; AAD: age at diagnosis; ZnT8: ZnT8-Ab titers.

In this study, the PC analysis was applied to a set of variables from a cohort of Tunisian T1D newly diagnosed children to identify a hierarchical clustering of multiple variables, correlated with various extents of T1D patterns. The 6 variables selected for PC analysis are presented in Table 4 which shows the stronger or weaker influence of, respectively, original variable on each of the two principal components. The PC-1 and -2 explained 23.52% and 20.7%, respectively, of the variation within the T1D dataset and reached both 44.20%. In PC-1, the dominant variables were ZnT8-Ab titers and AAD. Sera levels of GADA and AAD were the dominant variables in PC-2. The loading plot, with the relative position of each variable, is illustrated in Figure 2 by the orthogonal plane generated by the two PCs. Two patterns were evident: ZnT8-Ab titers and AAD mainly on the first PC while GADA titers and gender mainly on the second PC. IA-2A titers and genetic risk were equally distributed in PC-1 and PC-2 (Figure 2).

## 4. Discussion

In light of the knowledge that the determination of IAA at the onset of T1D is often biased by the administration of exogenous insulin [21], ICA, GADA, and IA-2A measurements were used for the diagnostic support of T1D in our laboratory. Indeed, our previous study reported that the serological biomarker offering the best benefit for testing T1D children is the GADA, alone or in combination with IA-2A and/or ICA [22]. Fifty years ago, ZnT8 was recognized as an autoantigen in T1D, and ZnT8-Ab introduction into the routine diagnostic process of T1D may improve the overall autoantibody sensitivity in different countries [5, 23]. Indeed, ZnT8-Ab was the only detectable marker of autoimmunity in 6/156 (3.84%) T1D newly diagnosed children, classified as auto-Ab negative in our study. This contribution to the diagnosis of T1D was higher than in previous studies, suggesting ZnT8-Ab as an additional biomarker to further characterize T1D with increased sensitivity [4, 24–27]. Moreover, our serological study found 42% Znt8-Ab+ in T1D newly diagnosed children. This result was not consistent with most of the studies performed in other countries. It was lower than that observed in the Caucasian population (60-80%) [4] and higher compared to Japanese patients at T1D onset [6]. ZnT8-Ab positivity was reported to be 65% in Argentinians with new-onset T1D [28] and 24% in Chinese T1D patients [29]. Alternatively, a Brazilian study that enrolled both a Caucasian and a non-Caucasian T1D population found a ZnT8-Ab+ prevalence of 24% in non-Caucasian participants. Also, Araujo et al. reported that neither ZnT8-Ab positivity nor level was associated with ethnicity [30], and in sub-Saharan Africa, there is considerable heterogeneity in the prevalence of auto-Ab profiles, and the impact of these immune markers in disease classification is still uncertain [27, 31]. Taking it together, ZnT8-Ab prevalence was associated with ethnicity in new-onset T1D children suggesting the modulated role of genetic factors [29, 32].

In addition to confirming that GADA is the most commonly detected Abs (70%) in Tunisian T1D children [33, 34], our serological study reported that the frequency and titers of ZnT8-Ab, IA-2A, and GADA are unrelated to gender, unlike ICA distribution which was higher in males compared to females. As the prevalence of ZnT8-Ab was found to be correlated with the onset of AAD in different studies [29, 35, 36], we investigated the AAD of ZnT8-Ab positive patients at diabetes onset. ZnT8-Ab seemed to be a better marker in teenagers (> 10 years of AAD) in comparison with children (under 10 years of AAD) with significantly higher titer. Our findings are consistent with a previous study that found that ZnT8-Ab positivity was associated with older age in children and adolescents younger than 15 years old and newly diagnosed with diabetes [37].

However, other studies in Chinese and Belgian patients with T1D reported that the prevalence of ZnT8-Ab declined with the increasing AAD [25, 29]. On the whole, it can be speculated that the AAD may play an important role in the ZnT8-Ab level but the correlation remains ambiguous, and the characteristics of population selection should be taken into consideration [24].

On the other hand, our study was per that of Salonen et al. confirming that children are mostly positive for multiple auto-Abs with a more frequent association of ZnT8-Ab plus IA-2A positivity, which was previously observed in new-onset T1D patients [36, 38]. Indeed, screening for IA-2A and ZnT8-Ab increases the diagnostic sensitivity for detection of autoimmunity as previously observed in new-onset T1D patients of five regional cohorts (Asia-Pacific, European, North American, Belgian, and the UK) [16, 32]. The authors suggested that IA-2A and ZnT8-Ab tend to cluster and predict, alone or in combination, rapid progression to diabetes, indicating that they preferentially mark the later stages of the preclinical phase of T1D [25]. It was sustained by the similarities between these two auto-Abs; IA-2A and ZnT8-Ab recognized both intracellular domains of their respective antigens [39–41], and they are integrated into the granule membrane itself [42]. These targets may only become accessible to the autoimmune response mechanisms after *β*-cell damage or dysfunction has occurred. Taking into account the role of environmental factors in the immune system activation in addition to genetics, the complex auto-Abs combination necessitated additional research.

Interestingly, there was a greater proportion of T1D children with AAD under 10 years without any Abs testing positive. The absence of IAA analysis was a limitation in our study because we could not know whether diabetes in this young age group presented this Ab or if it corresponded to monogenic diabetes. For that, HLA-typing should be tested to confirm the autoimmune origin of T1D in these children [43], and the extended family history of type 1 diabetes must be considered [44].

In light of our serological results, ZnT8-Ab could be a first-line screening marker along with ICA, GADA, and IA-2A for the diagnostic investigation of autoimmune diabetes in routine practice.

On the other hand, ZnT8-Ab is recognized as an epitope, the ZnT8 protein C terminal. Coding by the *SLC30A8* gene, this protein presents a polymorphic variant, rs13266634C/T, at the codon for the 325th amino acid conferring three ZnT8 protein isoforms (R325, W325, and rarely Q325). It seems associated with the occurrence of ZnT8-Ab [4]. Indeed, it has been demonstrated that the distribution of ZnT8R-Ab, ZnT8W-Ab, and ZnT8Q-Ab differs between populations [4, 5]. However, ZnT8-Ab variant distribution in Tunisian T1D children could not be determined, considering that the methodology used in our study detects Abs against all three of these ZnT8 variants. Several studies have focused on the role of *SLC30A8* rs13266634 polymorphisms in T1D patients from different populations, including our family-based association study in a Tunisian population [6, 17, 45]. Our result shows that the MAF of rs13266634 was similar to those observed in other populations, as well as the distribution of SLC30A8 genotype and allele among T1D patients and controls. Moreover, the transmission and the genetic models of rs13266634 C/T polymorphism are not heavily associated with T1D in newly diagnosed children. This finding is consistent with a meta-analysis study that found the C allele has no risk of developing T1D in family-based studies, and it is supported by a few studies in Danish, Japanese, and British populations [6, 46, 47]. However, a German case-control study showed higher frequencies of the C allele and CC genotype of the rs13266634 polymorphism in T1D patients than in controls [48].

Recently, Gomes et al. reported that an adjacent locus of rs2466293 in the *SLC30A8* gene may confer susceptibility to T1D in non-European descendants [38]. Our genetic study confirmed that the Tunisian samples have a certain genetic diversity due to the complex demographic history of migrations from within Africa, Europe, and the Middle East, despite their clustering together with the Berber groups from Morocco and Algeria and sharing a substantial genetic background [49, 50].

Using PC analysis to identify characteristic patterns in our dataset, we were able to dissect the heterogeneity of the T1D [19]. The analysis allows a correlation of variables into linear combinations, retaining most of them in a simplified cluster. Our analyses based on 6 demographic, immunological, and genetic variables identified two principal PC of the total variable clusters in the T1D dataset explaining 42% of the total variances, PC-1 and PC-2. PC-1 is in agreement with our experimental results and confirms our hypothesis of an existing relationship between ZnT8-Ab and the AAD in newly diagnosed children. That supports a clinically relevant implication of the ZnT8-Ab, especially in teenager T1D patients at disease diagnosis.

To our best knowledge, our study is the first focused on the prevalence of ZnT8-Ab in the North African population and demonstrated that it was similar to IA-2A and GADA frequencies. The ZnT8-Ab seems to constitute a hallmark of immune-mediated diabetes in Tunisian children and could have an added value in the detection of newly diagnosed children with T1D. However, our genetic results do not support an association between SLC30A8 rs1326634 and T1D in Tunisian children.

## Figures and Tables

**Table 1:** Demographic and clinical features of T1D Tunisian children's.

A.	*N*	%	Ratio
Sex			
Male	82	52.56	1.1
Female	74	47.43	
Family history of diabetes			
Yes	34	21.79	0.28
No	122	75.3	
B.		Median	SEM
Age at diagnosis (years)		7	±0.38
Glycemia at admission (mmol/l)		9.05	±0.52
Thyroid stimulating hormone (TSH)		2.37	±0.71
Hemoglobin A1c (HbA1c)		11.78	±2.48

Data are presented (A) as absolute numbers and percentages and (B) as median and standard error of the mean (SEM) in type 1 diabetes (T1D) Tunisian children.

**Table 2:** The effect of the inclusion of ZnT8-Ab assays in the panel of routinely auto-Abs on the sensitivity for beta-cell autoimmunity in type 1 diabetes (T1D) newly diagnosed children.

		Diabetic auto-Ab positive individual (1 auto-Ab)	Diabetic auto-Ab positive individual (≥1 auto-Ab)	Diabetic auto-Ab positive individual (≥2 auto-Ab)	Diabetic auto-Ab negative individuals
ICA, GADA, and IA-2A tested	No inclusion of ZnT8-Ab	61 (39.10%)	134 (85.90%)	73 (46.79%)	22 (14.10%)
	Inclusion of ZnT8-Ab	44 (28.21%)	140 (89.74%)	96 (61.54%)	16 (10.26%)
	*p* value	0.04	0.30	0.008	0.30

**Table 3:** Gender and prevalence of specific autoantibodies in ZnT8A positive (ZnT8-Ab+) and ZNT8-Ab negative (ZnT8-Ab-) in 156 type 1 diabetic (T1D) newly diagnosed children, based on the age at diagnosis (AAD).

	Children ≤ 5		6 ≤ children ≤ 10		Children ≤ 10		Children ≥ 11		*p* value
	ZnT8-Ab+	ZnT8-Ab-	ZnT8-Ab+	ZnT8-Ab-	ZnT8-Ab+	ZnT8-Ab-	ZnT8-Ab+	ZnT8-Ab-	ZnT8-Ab+
*N* (%)	25 (16.02)	31 (19.87)	30 (19.23)	20 (12.82)	55 (35.25)	51 (32.69)	32 (20.51)	18 (11.53)	0.037
Sex ratio (F/M)	1	1.18	1.09	1.4	1.04	1.2	0.56	0.42	—
ZnT8-Ab titer (UI/ml)	129.02 ± 55.13	1.00 ± 0.69	220.4 ± 91.29	1.8 ± 1.22	149.34 ± 73.21	1.27 ± 1.18	362.66 ± 87.73	0	0.009
GADA titer (UI/ml)	107.88 ± 54.68	287.2 ± 80.06	276.15 ± 88.11	349.00 ± 106.46	182.73 ± 72.39	307.8 ± 94.5	268.78 ± 86.68	419.70 ± 158.26	NS
IA-2A titer (UI/ml)	316.25 ± 166.99	162.97 ± 104.08	598.33 ± 185.42	334.88 ± 224.52	430.75 ± 176.15	220.28 ± 188.4	207.95 ± 114.59	201.17 ± 104.35	NS

T1D: type 1 diabetes; ZnT8-Ab: zinc transporter 8 autoantibodies; GADA: glutamic acid decarboxylase autoantibodies; IA-2A: antiprotein tyrosine phosphatase- (PTP-) like antigen IA-2 autoantibodies.

**Table 4:** Six clinical, immunological, and genetic T1D variables were included in PC analysis. The factor-loading matrix for two principal components illustrates the stronger or weaker influence of each variable on each component. Variables displaying an absolute factor loading ≥ 0.4 (in italic) were considered representative of each PC. A positive sign in the factor indicates that the PC was influenced by higher values of the original variable, while a negative sign indicates the influence of lower values.

Variable selected for PC analysis	Attribute name^a^	Extracted components	
		PC-1	PC-2
GADA titers	GAD	-0.038	*0.582*
IA-2A titers	IA2A	0.158	-0.258
ZnT8-Ab titers	ZnT8	*0.794*	-0.067
rs13266634 genotype	C/T	0.130	-0.719
Age at diagnosis	AAD	*0.691*	*0.497*
Gender	SEX	-0.509	0.263

^a^The attribute name is the same reported in Figure 2. ZnT8-Ab: zinc transporter 8 antibodies; GADA: glutamic acid decarboxylase antibodies; IA-2A: antiprotein tyrosine phosphatase- (PTP-) like antigen IA-2 antibodies.

## Data Availability

The data that support the findings of this study are available from the corresponding author upon reasonable request.

## Supplementary Materials

Supplementary Materials Supplementary Materials Results of the family-based association test (FBAT) analysis.
