# Supplementary material for: Autoantibodies to Zinc Transporter 8 and SLC30A8 Genotype in Type 1 Diabetes Childhood: A Pioneering Study in North Africa
Source: J Diabetes Res. 2022 May 23;2022:2539871. doi: 10.1155/2022/2539871 (PMC9152414; doi:10.1155/2022/2539871)
Supplement: Supplementary Materials — Results of the family-based association test (FBAT) analysis. [file 2539871.f1.docx]

**Supplementary Table:**

Results of the family-based association test (FBAT) analysis

| Locus | Allele/Genotype | Freq |
| --- | --- | --- |
| ZnT-8 gene  rs13266634 | A | 0.710 |
|  | C | 0.290 |
|  | AA | 0.526 |
|  | AC | 0.368 |
|  | CC | 0.106 |
